# “The Chennai declaration” - Indian doctors’ fight against antimicrobial resistance

**DOI:** 10.1186/2047-2994-2-7

**Published:** 2013-03-02

**Authors:** Andreas Voss, Abdul Ghafur

**Affiliations:** 1 Department of Medical Microbiology and Infectious Diseases, Radboud University Medical Centre and Canisius-Wilhelmina Hospital, Nijmegen, The Netherlands; 2 “Roadmap meeting” and “The Chennai Declaration”, Chennai based Consultant in Infectious Diseases and Clinical Microbiology, Chennai, India

## Abstract

“The Chennai Declaration” is the result of the first ever joint meeting of medical societies in India addressing antibiotic resistance. The declaration is not a policy by itself, but a call for a national policy. The Declaration has looked into all major aspects of the problem of antimicrobial resistance, has suggested practical solutions, explained in detail the responsibility of each and every stakeholder.

## 

From the start Antimicrobial Resistance and Infection Control had a sincere interest in global solutions to tackle the challenge of antimicrobial resistance [1,2]. Recently, the medical societies in India addressed the problem of antibiotic resistance, that deserves commentary and international endorsement [3].

## Commentary

Being the second most populous country in the world, any news on India, whether good or bad, echoes around the world. While this can be a privilege, the attention India received in the recent past with regard to NDM1 certainly was not. Indians felt discriminated for a problem that is global. While such sentiments in part seem justifiable from an emotional and political point of view, it is true, that the public health problem, is real. Indian hospitals have reported very high Gram-negative resistance rates (including very high rates of ESBL-producers and increasing rates of carbapenem resistance). In addition, the occurrence of resistance is not limited to health-care settings, but has widely spread into the environment and thus became a public-health problem. While the occurrence of ESBL and CRE is not unique to India, but truly global (from other countries on the Indian subcontinent to the USA), the scale of the problem on the Indian subcontinent is different, due to the alarming speed with which the resistance increases. The large population, sanitation issues, absence of functioning antibiotic policy, and inadequate infection control facilities in many hospitals make the region an epicentre of the spread of multi-drug-resistant micro-organisms, such as CRE’s.

“A roadmap to tackle the challenge of antimicrobial resistance”, was the first ever joint meeting of medical societies in India addressing antibiotic resistance, held in Chennai in August 2012 [3]. The high antimicrobial resistance rate in the country and the inspiration received from the on-going international efforts prompted Indian doctors to organise the Chennai meeting. Considering the large number of medical societies in India, ensuring participation of all major societies, as well as representatives from of governmental bodies such as the office of Drugs Controller General of India, Medical Council of India, National Accreditation Board of Hospitals, Indian Council of Medical Research, was a major achievement. The efforts resulted in a strategy of Indian medical societies and policy makers to combat the serious menace of antimicrobial resistance in the country.

“*The Chennai Declaration*” is the consensus evolved out of the meeting and co-authored by representatives of various medical societies [3]. The document is based on realistic goals and objectives, with a deep understanding of the background Indian scenario. Over the last decade or two, the Indian health-care infrastructure underwent significant changes. While possessing many world class corporate hospitals and institutes, the facilities available in many villages and remote areas are still vastly inadequate. Medication including antibiotics may be purchased over the counter and/or are prescribed by practitioners from alternative medical branches and healers. Formulating and implementing an antimicrobial stewardship program in one of the largest countries, with an enormously heterogenic and diverse health-care system, is indeed a huge challenge. Strict control on over the counter sale (OTC) as well as in hospital antibiotic usage should be the first steps of the policy. Whether such a policy is implementable on the Indian subcontinent is an issue that warrants serious debate. The lack of (qualified) doctors in many remote places possibly makes the complete ban of OTC antibiotics throughout the country obsolete. Consequently, a targeted strategy of absolute control in densely populated areas, where qualified doctors will be available, and a more liberal approach in remote places, with monitoring of a selected list of oral antibiotics, should be more feasible.

In health-care systems like those in the United Kingdom’s NHS or the Dutch hospitals, clinical microbiologists in co-operation with pharmacists and infectious diseases specialists can enforce tight control of antibiotic usage in hospitals. Though there is reasonably good availability of microbiologists in most parts of India, the lack of adequate clinical orientation in undergraduate and post graduate microbiology training and the lack of interaction between clinicians and microbiologists need to be properly addressed and necessary curriculum changes to be made to rectify the deficits. The country also needs a good number of infectious diseases physicians. Overall, it is of importance that all specialities in and around the diagnostic, prevention and treatment of infectious diseases work together. The Dutch foundation for antibiotic policies (SWAB) recently formulated a guideline that by January 2014 all hospitals need to have such a multidisciplinary team, referred to as “A-team”. Last but not least, the last stakeholder in the filed, the pharmaceutical industry, should be involved. Major companies supported unrestricted grants for antimicrobial stewardship projects thereby showing their involvement. Unfortunately this grants are frequently restricted to the US, and should be extended to “growing markets” like India.

“The Chennai Declaration” is a not a policy by itself, but a call for a national policy. The Declaration has looked into all major aspects of the problem, has suggested practical solutions, explained in detail the responsibility of each and every stakeholder. The Declaration is an outcry for action by the medical community and the authorities, not another publication on antimicrobial resistance waiting to get shelved!

The Chennai Declaration” is available from: http://www.indianjcancer.com/preprintarticle.asp?id=104065.
